# How accurate are geriatricians’ fall predictions?

**DOI:** 10.1186/s12877-022-03129-w

**Published:** 2022-05-18

**Authors:** Jason Wilbur, Gerald Jogerst, Nicholas Butler, Yinghui Xu

**Affiliations:** Department of Family Medicine, Roy J. and Lucille A. Carver College of Medicine, University of Iowa, Iowa City, Iowa, USA

**Keywords:** Older adults, Fall risk, Prediction, Screening

## Abstract

**Background:**

Older patients are at increased risk of falling and of serious morbidity and mortality resulting from falls. The ability to accurately identify older patients at increased fall risk affords the opportunity to implement interventions to reduce morbidity and mortality. Geriatricians are trained to assess older patients for fall risk. If geriatricians can accurately predict fallers (as opposed to evaluating for individual risk factors for falling), more aggressive and earlier interventions could be employed to reduce falls in older adult fallers. However, there is paucity of knowledge regarding the accuracy of geriatrician fall risk predictions. This study aims to determine the accuracy of geriatricians in predicting falls.

**Methods:**

Between October 2018 and November 2019, a convenience sample of 100 subjects was recruited from an academic geriatric clinic population seeking routine medical care. Subjects performed a series of gait and balance assessments, answered the Stay Independent Brochure and were surveyed about fall incidence 6–12 months after study entry. Five geriatricians, blinded to subjects and fall outcomes, were provided the subjects’ data and asked to categorize each as a faller or non-faller. No requirements were imposed on the geriatricians’ use of the available data. These predictions were compared to predictions of an examining geriatrician who performed the assessments and to fall outcomes reported by subjects.

**Results:**

Kappa values for the 5 geriatricians who used all the available data to classify participants as fallers or non-fallers compared with the examining geriatrician were 0.42 to 0.59, indicating moderate agreement. Compared to screening tools’ mean accuracy of 66.6% (59.6–73.0%), the 5 geriatricians had a mean accuracy for fall prediction of 67.4% (57.3–71.9%).

**Conclusions:**

This study adds to the scant knowledge available in the medical literature regarding the abilities of geriatricians to accurately predict falls in older patients. Studies are needed to characterize how geriatrician assessments of fall risk compare to standardized assessment tools.

## Background

Worldwide, the percentage of persons over the age of 60 years is growing faster than any other age group, and fall-related injuries are a major source of morbidity and mortality for older adults, accounting for 40% of all injury-related deaths [1]. In 2018 in the United States, 27.5% of adults 65 years of age and older reported at least one fall in the previous year, and 10.2% reported at least one fall-related injury [2]. Health care spending on older adult falls in the U.S. is estimated to be $50 billion per year [3].

While falls are highly prevalent in older adults, they also represent a preventable source of morbidity, mortality and cost to the health care system. If older adult fallers can be identified prior to an injurious fall, multifactorial risk factor assessment and intervention may prevent falls [4]. However, over 100 fall risk assessment tools have been developed and published without a clear “gold standard” emerging [5]. Given their clinical training, which includes fall risk assessment, geriatricians may be anticipated to possess a high degree of accuracy for predicting fall risk in older adults. However, there is paucity of data to either support or refute this assertion, and a search of the medical literature reveals no publications directly comparing geriatricians’ clinical gestalt for fall risk with more objective screening tools [6]. The purpose of this study is to compare the geriatricians’ predictions of faller/non-faller to (a) comprehensive assessment by a single examining geriatrician reference standard and (b) participant falls reported at 6–12 months after study entry.

## Methods

A convenience sample of 100 participants was recruited from an undifferentiated older population followed for routine medical care during their visit in an academic geriatric clinic. After obtaining informed consent from the participant and/or caregiver in cases of cognitive impairment, RunScribe™ inertial measurement units (IMU) were attached to each subject’s shoes in the mid-foot location and to the posterior collar at the approximate C-7 mid-line level [7]. After the subjects were instructed in the performance of the clinical assessment tests using the Centers for Disease Control and Prevention (CDC) initiative, they completed 4-Stage Balance, Timed Up and Go (TUG) and 30 Second Chair Stands tests, in that order [8]. The examining geriatrician and assistant stood on either side of the subject during the tests for safety, and no subjects fell during the procedures. The research assistant used iPhone applications to upload IMU data and keep times during the testing. All subjects completed the Stay Independent Brochure (SIB) during their visit.

After the subject departed the clinic, the examining geriatrician reviewed the subjects’ electronic medical record and recorded the subjects’ age, gender, height, weight, body mass index, blood pressure, number of chronic medical conditions documented on the problem list, movement disorder diagnoses (e.g., parkinsonism, restless leg syndrome), number of chronic scheduled medications, number of psychotropic medications (any centrally acting medications taken daily, e.g., anti-depressants, anti-psychotics) and entered these data into an Excel file, along with all the clinical assessment test results. After evaluating all clinical data that would be collected during a clinic visit for a fall evaluation, the examining geriatrician assigned the subject as a faller or non-faller. All subjects were sent questionnaires 6 to12 months (mean for all subjects was 9 months) following clinic evaluation to query if they had fallen since the initial evaluations. The inertial measurement data were stored for use in a follow-up experiment reported elsewhere [9].

Independent variables included in analyses were: SIB (0–12), 4 Stage Balance (4–40 sec), TUG (7–123 sec), 30 Second Chair Stands (0–23). The examining geriatrician’s designation of faller vs non-faller was used as the dependent variable for the above independent variables and as an independent variable when comparing it with the prospective data of fall reports from follow-up questionnaires.

All clinical data were shared with 5 board certified geriatricians with a combined clinical experience of 100 years, ranging from 4 to 35 years post-fellowship. They were instructed to use these data to classify the participants as fallers versus non-fallers. They were given no additional instructions and were free to weigh the data provided as they saw fit. These geriatricians were not present during the evaluations and were blinded to the examining geriatrician’s fall risk designations and to the fall outcomes of the subjects. Kappa values were calculated between each of these 5 geriatricians and the examining geriatrician (reference standard). All six geriatricians’ assessments were checked for reliability as well as accuracy of predicting future falls reported on follow-up participant questionnaire.

Standard descriptive statistics were used to summarize the variables. The Pearson chi-square test was used to compare discrete variables, and t-test was used to compare continuous variables between two groups. When the outcome variable was not normally distributed, the Wilcoxon sum-rank test was used. The kappa statistic was conducted to assess agreement between the two dichotomous variables. Sensitivity, specificity, positive and negative likelihood ratios, positive and negative predictive values, and overall accuracy were calculated with 95% confidence intervals, using the method described by Simel et al. [10]. All analyses were performed using SAS version 9.4 (SAS Institute Inc., Cary, NC).

## Results

One-hundred subjects with a mean age of 75 years (65–96 years), 51 males, completed the study between October 2018 and November 2019. Fifty-four participants were classified as fallers by the examining geriatrician and 25 of 89 (28%) subjects who returned the follow-up questionnaire reported any fall occurring 6 to 12 months following study entry.

Table 1 shows the characteristics of the 100 subjects classified as fallers or non-fallers by the examining geriatrician. Among the subjects, there were 10 diagnoses of cognitive impairment, 3 of Parkinson disease, 4 of peripheral neuropathy, 20 of lower extremity orthopedic conditions, 5 of brain injury, and 9 of vestibular and/or hearing impairments. There were significant associations between the examining geriatrician’s classification of faller/non-faller with the following subject characteristics: number of diagnoses; number of medications; SIB score; and the clinical tests of 4 Stage Balance, TUG and 30 second chair stands.

**Table 1:** Characteristics of Subjects classified by geriatrician’s assessment

	Total *N =* 100	Assigned as Fallers *N =* 54	Assigned as Non-Fallers *N =* 46	*p-*values
Mean Age (years)	75.4	77.5	72.9	0.003
%Female Gender	49	57	39	0.068^a^
Mean BMI	28.8	29.9	27.5	0.038
Mean BP systolic/diastolic	136/76	137/76	134/76	0.404/0.752
Mean Number of Diagnoses	8.4	10.1	6.3	<.0001
Mean Number of Movement Disorders	0.4	0.6	0.2	0.009^b^
Mean Number of Medications	7.7	9.4	5.7	<.0001
Mean Number of Psychoactive Medications	0.9	1.2	0.5	0.012^b^
SIB (score)	3.3	5.1	1.2	<.0001
4 stage balance (seconds)	31.1	26.1	37.0	<.0001
TUG (seconds)	14.7	17.7	11.2	<.0001^b^
Mean Number of 30 second Chair Stands	10.3	8.3	12.8	<.0001

^a^Chi-square test; ^b^Wilcoxon rank-sum test; others are t-tests

*SIB* Stay Independent Brochure, *TUG* Timed-Up-and-Go, *BMI* Body Mass Index, *BP* Blood pressure

Table 2 describes the characteristics of the 89 subjects who returned the follow-up questionnaire. There were significant associations between subjects reporting falling and the number of diagnoses, SIB score and 4 Stage Balance test.

**Table 2:** Characteristics of Subjects classified by follow-up fall reports

	Total *N =* 89	Reported as Fallers *N =* 25	Reported as Non-Fallers *N =* 64	*p-*values
Mean Age (years)	75.3	76.8	74.7	0.234
%Female Gender	49	44	52	0.521^a^
Mean BMI	28.8	28.0	29.1	0.426
Mean BP systolic/diastolic	135/76	136/75	134/76	0.815/0.619
Mean Number of Diagnoses	8.4	9.9	7.9	0.023
Mean Number of Movement Disorders	0.5	0.5	0.5	0.966^b^
Mean Number of Medications	7.7	8.5	7.4	0.236
Mean Number of Psychoactive Medications	0.9	1.2	0.8	0.154^b^
Mean SIB (score)	3.2	4.7	2.7	0.014
Mean 4 stage balance (seconds)	31.4	28.2	32.6	0.022
Mean TUG (seconds)	14.1	17.6	12.8	0.101^b^
Mean Number of 30 second Chair Stands	10.6	9.2	11.1	0.064

^a^Chi-square test; ^b^Wilcoxon rank-sum test; others are t-tests

*SIB* Stay Independent Brochure, *TUG* Timed-Up-and-Go, *BP* Blood Pressure, *BMI* Body Mass Index

Table 3 compares the clinical screening tools and geriatricians’ prediction of falls to the outcome of subject-reported falls. The highest accuracy of the clinical tools was 73% for the 30 Second Chair Stand test. Sensitivities for the clinical tests were approximately 50%. The examining geriatrician had the highest sensitivity (76%) for predicting falls, with an accuracy of 61%. The other geriatricians had a sensitivity for predicting falls around 50%, with a maximum accuracy of 72%.

**Table 3:** Screening Tools and Geriatricians’ Prediction of Fallers at Follow-up

	Sensitivity (95% CI)^a^	Specificity (95% CI)	Positive LR (95% CI)	Negative LR (95% CI)	Positive PV (95% CI)	Negative PV (95% CI)	Accuracy (95% CI)
SIB	56.0 (34.9–75.6)	70.3 (57.6–81.1)	1.89 (1.13–3.15)	0.63 (0.39–1.00)	42.4 (25.5–60.8)	80.4 (67.6–89.8)	66.3 (55.5–76.0)
4-Stage Balance	48.0 (27.8–68.7)	64.1 (51.1–75.7)	1.34 (0.79–2.26)	0.81 (0.53–1.23)	34.3 (19.1–52.2)	75.9 (62.4–86.5)	59.6 (48.6–69.8)
TUG	52.0 (31.3–72.2)	73.4 (60.9–83.7)	1.95 (1.12–3.40)	0.65 (0.42–1.01)	43.3 (25.5–62.6)	79.7 (67.2–89.0)	67.4 (56.7–77.0)
30 sec chair stands	52.0 (31.3–72.2)	81.3 (69.5–89.9)	2.78 (1.47–5.24)	0.59 (0.39–0.90)	52.0 (31.3–72.2)	81.3 (69.5–89.9)	73.0 (62.6–81.9)
Examining Geriatrician’s Fall Risk Rating	76.0 (54.9–90.6)	54.7 (41.8–67.2)	1.68 (1.18–2.38)	0.44 (0.21–0.91)	39.6 (25.8–54.7)	85.4 (70.8–94.4)	60.7 (49.8–70.9)
Geriatrician 2	56.0 (34.9–75.6)	71.9 (59.2–82.4)	1.99 (1.18–3.36)	0.61 (0.38–0.98)	43.8 (26.4–62.3)	80.7 (68.1–90.0)	67.4 (56.7–77.0)
Geriatrician 3	52.0 (31.3–72.2)	79.7 (67.8–88.7)	2.56 (1.39–4.73)	0.60 (0.39–0.92)	50.0 (29.9–70.1)	81.0 (69.1–89.8)	71.9 (61.4–80.9)
Geriatrician 4	56.0 (34.9–75.6)	75.0 (62.6–85.0)	2.24 (1.29–3.88)	0.59 (0.37–0.93)	46.7 (28.3–65.7)	81.4 (69.1–90.3)	69.7 (59.0–79.0)
Geriatrician 5	52.0 (31.3–72.2)	59.4 (46.4–71.5)	1.28 (0.79–2.07)	0.81 (0.51–1.27)	33.3 (19.1–50.2)	76.0 (61.8–86.9)	57.3 (46.4–67.7)
Geriatrician 6	48.0 (27.8–68.7)	79.7 (67.8–88.7)	2.36 (1.25–4.46)	0.65 (0.44–0.97)	48.0 (27.8–68.7)	79.7 (67.8–88.7)	70.8 (60.2–80.0)
*N =* 89 Reported Fallers = 25							

^a^Simel DL, Samsa GP, Matchar DB. Likelihood ratios with confidence: sample size estimation for diagnostic test studies. J Clin Epidemiol 1991;44(8): 763–770

*CI* Confidence intervals, *SIB* Stay Independent Brochure, *TUG* Timed-Up-and-Go, *LR* Likelihood ratio, *PV* Predictive value

The kappa values for the 5 blinded geriatricians compared to the reference geriatrician ranged from 0.42 to 0.59, indicating moderate agreement [11]. The pooled mean and median accuracy of faller/non-faller assignment of the 5 blinded geriatricians was 67.4 and 69.7%, respectively.

## Discussion

Falls are the leading cause of unintentional injury death in older adults in the U.S., and the age-adjusted mortality rate of falls is rising [12]. If it can be done accurately, the identification of older adults at risk of falling has the potential to reduce morbidity and mortality in this population through multifactorial interventions [4].

With over 100 fall risk assessment tools in existence, including performance-based assessments and self-administered questionnaires, the sheer number belies any assertion of a “gold standard” test for determining a patient’s risk of falling [5]. An ideal tool would accurately place patients into high fall risk or low fall risk categories. At a minimum, a good screening test should possess a high degree of sensitivity, so as not to miss fallers [13]. However, at least one systematic review concluded that none of the currently available fall risk assessment tools possess sufficient validity [14].

Since the American Geriatrics Society and British Geriatrics Society practice guideline recommends multifactorial assessment of fall risk for patients presenting with a fall or gait/balance difficulties, we used the examining geriatrician’s assessment as our reference standard [15]. The examining geriatrician had a higher sensitivity than any of the other geriatricians but slightly lower accuracy for predicting falls. None of the standardized screening tools was as sensitive as the examining geriatrician for predicting fall risk.

The examining geriatrician used all the data available to assign a fall prediction, relying strongly on the number of diagnoses and medications as well as the SIB score, 4 stage balance test, TUG and 30 second chair stands (as indicated by the *p-*values for these associations; see Table 1). The number of diagnoses, SIB score and 30 second chair stands remained significantly related to falls prediction at follow-up (Table 2).

Since fall risk in older adults is dependent on many variables, some of which change rapidly (e.g., environment, blood pressure), one might reasonably ask the question, “What is the maximum degree of accuracy that can be expected from any fall risk assessment?” According to Rubenstein, approximately 30% of falls are caused by accidents and environmental factors [16]. In geriatrics parlance, these are often referred to as extrinsic risk factors, as opposed to intrinsic risk factors (i.e., risk factors intrinsic to the patient). In a clinical setting, a geriatrician applying either objective screening tests or clinical gestalt is likely to miss extrinsic risk factors. The geriatrician will only be able to assess such extrinsic factors as footwear and assistive device use, missing obstacles, tripping hazards and other fall risks in the patient’s environment. Because of the clinical setting, the geriatrician will focus on intrinsic risk factors, such as age, fall history, musculoskeletal problems, and gait abnormalities.

Additionally, some risk factors for falls are dynamic, changing over time. In the office or hospital setting, a geriatrician will only have a snapshot of the patient, incompletely assessing dynamic risk factors, such as blood pressure, gait, balance, medication use, and fear of falling.

Since any clinically administered fall risk assessment will rely almost exclusively on the evaluation of intrinsic risk factors in a single snapshot, and if Rubenstein is correct, perhaps the maximum accuracy of fall risk prediction in this setting is around 70%. If that is the case, our pooled mean geriatricians’ predictive accuracy of 67.4% approaches the ideal.

Limitations of this study include a sample that was drawn from a single academic center, a small cohort of geriatricians working in the same academic center and recall bias of the subjects who were queried regarding falls. Our study can only be applied to geriatricians evaluating community-dwelling older adults. The outcome of greatest interest to clinicians is injurious falls, which are less frequent and therefore harder to study than all falls. As with most studies of falls in older adults, ours is limited by necessary use of the surrogate endpoint of any fall as opposed to injurious fall.

## Conclusions

This study adds to the scant published knowledge of the accuracy of geriatrician fall-risk assessment. In this study, it appeared that geriatricians applied differing weights to fall risk factor data in assigning fall predictions. There was only moderate agreement between the examining geriatrician and the other geriatricians who used the data only to assign the fall prediction. However, due to extrinsic and dynamic risk factors, a geriatrician’s ability to predict future falls may be limited to a best accuracy of 70%. For this reason, frequent re-evaluations are needed in those at highest risk of falling.

Further studies are needed to better characterize the accuracy of fall risk predictions by geriatricians and other clinicians and to assess clinicians’ accuracy for predicting injurious falls. Additionally, comparisons between clinically administered fall risk assessments versus home or publicly administered assessments by trained fall risk screeners may help to elucidate the importance of extrinsic fall risk factor assessment. Finally, geriatricians (and other clinicians) are in a unique position to provide patient-centered, individualized assessments of fall risk, as called for by the global initiative currently developing new international guidelines to reduce falls in older adults [17]. More studies like ours will be needed to inform these and other fall reduction initiatives.

## Data Availability

All data generated or analyzed during this study are included in an attached supplementary MS Excel file.
